# Cognitive and Emotional Peri‐Implant Diseases Perception in a Cohort of Periodontitis Patients: A University‐Based Cross‐Sectional Study

**DOI:** 10.1111/jcpe.70011

**Published:** 2025-08-18

**Authors:** Isabella De Rubertis, Adriano Fratini, Alice Ferrari, Raffaele Mirra, Nicola Discepoli

**Affiliations:** ^1^ Unit of Periodontics, Department of Medical Biotechnologies University of Siena Siena Italy; ^2^ Private Practice Perugia Italy

**Keywords:** dental implants, perception, prevention, psychometrics, surveys and questionnaires

## Abstract

**Background and Aim:**

Patients' disease perception plays a fundamental role in adherence to therapy and participation in long‐term maintenance programmes. This cross‐sectional study aimed to assess the cognitive and emotional representation of peri‐implant diseases (PIDs) in a cohort of patients with periodontitis.

**Methods:**

Patients diagnosed with both periodontitis and PIDs were enrolled. Psychometric evaluation was conducted using the Brief Illness Perception Questionnaire (Brief‐IPQ) and the Oral Health Impact Profile‐14. Differences were analysed based on disease severity, and a multivariate logistic regression model was applied to explore associations between patient and implant‐level variables and illness perception.

**Results:**

A total of 148 patients (459 implants) were included. Patients exhibited low perception of PIDs, with no differences between peri‐implant mucositis and peri‐implantitis. However, participants with both peri‐implantitis and stage III/IV periodontitis presented with significantly higher Brief‐IPQ scores. Having ≥ 3 implants and a history of periodontitis treatment were associated with a 4.16‐fold and 6.58‐fold increase, respectively, in the odds of a higher Brief‐IPQ score.

**Conclusions:**

The cognitive and emotional representation of PIDs was found to be low among patients with both periodontitis and PIDs, but appeared to increase in more advanced clinical profiles. A higher number of implants and prior periodontal treatment were associated with greater disease representation.

**Trial Registration:** The study protocol was approved by the University Hospital of Siena Ethics Committee (Siena, Italy) (Sezione Area vasta Toscana Sud Est, no. 25906), and it was registered on Clinicaltrials.gov (registration number: NCT06383351)

## 1 Introduction

Peri‐implant diseases (PIDs) are inflammatory conditions that affect tissues surrounding dental implants, primarily caused by the accumulation of peri‐implant plaque biofilm (Berglundh et al. 2018). Their prevalence ranges from 19% to 65% for peri‐implant mucositis, and from 1% to 47% for peri‐implantitis (Derks and Tomasi 2015; Salvi et al. 2019). Prevention and successful management of peri‐implant biological complications require consistent adherence to self‐care regimens and regular attendance at recall visits (Carra et al. 2023; Ferreira et al. 2006; Frisch et al. 2020; Serino and Ström 2009). However, the effectiveness of supportive peri‐implant therapy (SPIT) is influenced by the patient's level of compliance, which, according to available literature, is generally low (Amerio et al. 2020). Indeed, scarce information and motivation are often the main reasons for unsatisfactory levels of compliance (Amerio et al. 2020).

Oral health literacy (OHL) is defined as the ability of individuals to understand and process oral health information, which consequently influences how they perceive their oral health status (Holtzman et al. 2017; Roundtable on Health Literacy, Board on Population Health and Public Health Practice, & Institute of Medicine 2013). Realistic disease representations determine positive oral health outcomes, as they lead to proactive behaviours, such as sticking to good oral hygiene practices and preventive care (IOM, Institute of Medicine 2013; Petrie and Weinman 2006). The Illness Perception Questionnaire (IPQ) and its brief version, the Brief Illness Perception Questionnaire (Brief‐IPQ), were developed to assess how patients conceptualise their disease. Brief‐IPQ is derived from Leventhal's Common‐Sense Model of Self‐Regulation (CSM) and evaluates patients' perceptions about the identity, cause, timeline and consequences of their illness (Leventhal et al. 2016; Weinman et al. 1996).

Patients' perceptions of periodontitis and its treatment have been thoroughly investigated (De Pinho et al. 2012; Durham et al. 2013; Ferreira et al. 2017; Jansson et al. 2014) and the significant influence of periodontitis on oral health–related quality of life has been documented (Graziani and Tsakos 2020). Studies have shown that patients with more advanced stages of periodontitis experience significantly greater impacts on their quality of life. This is reflected by higher psychosocial impact and increased disability weights, which are quantitative estimates of disease burden derived from health state evaluations (Brennan et al. 2007). Such measures highlight the disproportionate impact of severe stages of periodontitis compared to earlier ones (Brennan et al. 2007; Nisanci Yilmaz et al. 2022; Buset et al. 2016). Moreover, recent studies have demonstrated the predictive value of certain domains of the Brief‐IPQ on patient adherence to periodontal therapy and their impact on treatment effectiveness (Discepoli et al. 2022; Machado et al. 2020).

Given the lack of available data on the perception of PIDs, and considering that patients with periodontitis are known to experience a higher disease burden and a greater impact on the quality of life, the present study focused on individuals diagnosed with both conditions.

Therefore, the primary objective was to evaluate the cognitive and emotional representation of PIDs in a cohort of patients affected by both periodontitis and PIDs. Additionally, we aimed to identify the influence of patient and implant‐level variables in shaping illness representation and to describe patient‐reported outcomes related to the quality of life within the study cohort.

## 2 Materials and Methods

### 2.1 Study Design

The present cross‐sectional study is reported according to the Strengthening the Reporting of Observational Studies in Epidemiology (STROBE) guidelines (Vandenbroucke et al. 2007; von Elm et al. 2008).

The investigation adhered to the principles outlined in the Declaration of Helsinki. All enrolled subjects were informed about the study protocol and were asked to read and sign the informed consent form.

### 2.2 Setting and Participants

From April 2024 to June 2024, patients with suspected PID requiring comprehensive diagnostic assessment were consecutively recruited at the Unit of Periodontics, Department of Medical Biotechnologies, ‘AOUS, Le Scotte’ University Hospital (Siena, Italy). All eligible patients presenting during this period underwent a standardised evaluation protocol.

Eligible patients were required to meet the following inclusion criteria: (i) age between 18 and 80 years; (ii) diagnosis of periodontitis; (iii) presence of at least one implant, loaded 1 year prior to their referral; (iv) presence of bleeding and/or suppuration after gentle probing of the peri‐implant mucosa (> 1 spot of bleeding; Tonetti et al. 2023); (v) absence of systemic conditions that are uncontrolled or may interfere with oral or systemic health–related behaviours, healing or the ability to participate in psychometric assessments (e.g., uncontrolled diabetes, unstable cardiovascular disease, active cancer, or severe cognitive/psychiatric disorders) and (vi) the ability to give written informed consent.

Patients with one or more of the following characteristics were excluded from the study: (i) pregnant or lactating women; (ii) inability to communicate in Italian; or (iii) inability to perform oral hygiene manoeuvres.

None of the participants had previously been treated at the centre where the study was conducted.

### 2.3 Screening and Anamnesis

Participants were screened for periodontitis through a comprehensive objective examination of all tooth surfaces. Periodontal cases were defined according to the 2018 Classification and Case Definition (Tonetti et al. 2018).

Data about patients' age, gender, smoking status, body mass index (BMI), presence of comorbidities and information regarding prior treatment for periodontitis or PID elsewhere were recorded.

### 2.4 Psychometric Variables

After the initial screening phase, patients were asked to understand and fill the Brief Illness Perception Questionnaire (Brief‐IPQ) (Broadbent et al. 2006) and the Oral Health Impact Profile 14 (OHIP‐14) (Slade 1997), in their validated Italian versions (Pain et al. 2006; Corridore et al. 2014).

#### 2.4.1 Brief‐IPQ

The Brief‐IPQ (Broadbent et al. 2006) consists of three domains: ‘cognitive illness’ (domain 1), ‘emotional representation’ (domain 2) and ‘illness comprehensibility’ (domain 3). The three sections explore different psychometric dimensions and provide information about the patient's disease representation, assessed through nine items. Domain 1 includes the following items: ‘consequences’ (item 1), ‘timeline’ (item 2), ‘personal control’ (item 3), ‘treatment control’ (item 4) and ‘identity’ (item 5). ‘Concern’ (item 6) and ‘emotional response’ (item 8) belong to domain 2. Domain 3 is represented by a single question: ‘understanding’ (item 7). Responses are recorded on a Likert‐type scale ranging from 1 to 10.

#### 2.4.2 OHIP‐14

OHIP‐14 is composed of seven domains (functional limitation, physical pain, psychological discomfort, physical disability, psychological disability, social disability and handicap), which comprise a total of 14 questions. Responses in the OHIP‐14 are recorded using a Likert‐type scale from 0 to 4 (Slade 1997). For both the Brief‐IPQ and OHIP‐14 questionnaires, individual item responses can be summed to obtain a total score. Higher total scores indicate, respectively, a greater disease perception (Brief‐IPQ) and a more significant impact on oral health–related quality of life (OHRQoL).

### 2.5 Clinical Variables

Clinical examination was performed by two trained and calibrated examiners (A.F., I.D.R.). Intra‐examiner reliability was assessed for each examiner using the intra‐class correlation coefficient (ICC = 0.96 and 0.94, respectively), based on repeated probing pocket depth (PPD) measurements performed on four non‐study subjects diagnosed with peri‐implantitis. ICCs were calculated using a two‐way mixed‐effects model with absolute agreement. Inter‐examiner agreement was evaluated using Cohen's Kappa (κ = 0.90), also based on PPD measurements at peri‐implant sites. Patients underwent a full‐mouth periodontal and peri‐implant examination, using a manual UNC‐15 periodontal probe (PCP15; Hu‐Friedy, Chicago, IL, USA) at six sites per tooth/implant (Lang et al. 1991). Full‐mouth plaque score (FMBS) and bleeding score (FMPS) were calculated as the proportion of tooth and implant surfaces bleeding and presenting plaque upon probing (O'Leary et al. 1972; Tonetti and Claffey 2005). Periodontitis' stage and grade were assigned according to the current classification (Tonetti et al. 2018).

At the implant level, recession was measured as the distance from the prosthetic margin to the peri‐implant mucosa margin. Keratinised tissue width (KTW) was determined as the distance between the mucosal margin and the mucogingival junction in a mid‐facial position (Souza et al. 2016). Presence of suppuration (SUP) was assessed at six sites per implant. Moreover, details about implant‐specific features were recorded. The diagnosis of PID was made according to the 2018 case definition and the ID‐COSM initiative consensus (Berglundh et al. 2018; Tonetti et al. 2023).

### 2.6 Radiographic Variables

In the absence of previous good‐quality radiographic records, a periapical intraoral radiograph was taken for each implant to evaluate marginal bone levels using the long‐cone paralleling technique. Peri‐implantitis was identified by bone loss of ≥ 3 mm apical to the most coronal portion of the intraosseous part of the implant (Berglundh et al. 2018; Tonetti et al. 2023).

### 2.7 Study Size

At the time of study design, no published data were available assessing similar psychometric outcomes in a comparable population. Therefore, a conventional a priori sample size calculation based on expected effects for psychometric variables was not feasible.

Instead, the sample size estimation was based on the expected prevalence of peri‐implantitis, using reference data from Derks and Tomasi (2015), which reported a prevalence of 45% (Derks and Tomasi 2015). This value was adjusted to 60% to reflect the typically higher prevalence of peri‐implantitis observed in university‐based settings, as suggested by Vignoletti et al. (2019). Assuming a significance level of 0.05 (*α*) and a desired power of 90% (corresponding to a Type II error *β* of 10%), the estimated required sample size was 148 participants. This estimation served as a pragmatic recruitment target, aimed at enrolling a sufficiently representative population of periodontal patients in which to explore the cognitive representation of PIDs.

To further support the robustness of the study findings, a post hoc power analysis was conducted based on the final logistic regression model. This analysis confirmed a statistical power of 0.94, supporting the adequacy of the sample in detecting the associations of interest. Appendix 1 gives detailed information on the post hoc power analysis.

### 2.8 Statistical Methods

Analyses were conducted using a dedicated software (STATA IC, version. 18, StataCorp LP, TX, USA).

Continuous data were expressed as mean and standard deviation, and categorical variables were reported as count and proportion. Given their ordinal nature, responses for each item and sum scores of the questionnaires (both the Brief‐IPQ and the OHIP‐14) were reported as median and interquartile range (IQR). The normality of data distribution was evaluated with the Shapiro–Wilk test. The Mann–Whitney U test and the Kruskal–Wallis test were applied to assess differences in both questionnaires according to the diagnosis of PID and periodontitis. A multivariate logistic regression model was constructed at the patient level, with implant‐level variables aggregated per patient. The Brief‐IPQ sum score was dichotomised (cut‐off = 28, corresponding to the cohort median) and introduced as the dependent variable. Covariates were selected using Stata ‘allsets’ command, and the final model was run using the ‘logistic’ command. Some independent variables were also dichotomised based on the median of their distributions.

## 3 Results

A total of 154 individuals were assessed for eligibility and 148 of them met the inclusion criteria and agreed to participate in the current investigation. Six patients, each with a single implant functionally loaded (i.e., in prosthetic function) for less than 1 year, were excluded. Ultimately, a final sample of 148 patients (459 implants) was included.

### 3.1 Demographic and Clinical Variables

Peri‐implant mucositis was diagnosed in 65% of participants (*n* = 96), while peri‐implantitis was identified in the remaining 35% (*n* = 52).

Patients' age ranged from 31 to 79 years (mean 60.68 ± 9.26 years); the majority were women (60.81%), and 33.78% of them reported that they underwent periodontitis treatment elsewhere in the past. Regarding socioeconomic characteristics, the majority (46.62%) declared having high school education and reported being employed (58.11%) or retired (31.76%). Most of the participants were married (55.41%). All comorbidities reported by participants were recorded. Among these, only cardiovascular disease (15.54%) and diabetes (13.51%) were present in the study cohort, as detailed in Table 1.

**TABLE 1:** Patient demographic and clinical variables.

	Overall (*N* = 148)	Peri‐implant mucositis (*N* = 96)	Peri‐implantitis (*N* = 52)
Gender (*N* [%])			
Male/Female	58 (39.19)/90 (60.81)	38 (39.58)/58 (60.42)	20 (38.46)/32 (61.54)
Age (years) (Mean [SD])	60.68 (9.26)	59.72 (8.94)	62.46 (9.67)
History of periodontitis treatment (*N* [%])	50 (33.78)	31 (32.29)	19 (36.54)
Education (*N* [%])			
Elementary school	4 (2.70)	1 (1.04)	3 (5.77)
Middle school	30 (20.27)	21 (21.88)	9 (17.31)
High school	69 (46.62)	43 (44.79)	26 (50.00)
University	45 (30.41)	31 (32.29)	14 (26.92)
Occupation (*N* [%])			
Employed	86 (58.11)	57 (59.38)	29 (55.77)
Unemployed	10 (6.76)	6 (6.25)	4 (7.69)
Retired	52 (35.14)	33 (34.38)	19 (36.54)
Marital status			
Never married/Single	8 (5.41)	6 (6.25)	2 (3.85)
Married	82 (55.41)	56 (58.33)	26 (50.00)
Divorced	26 (17.57)	18 (18.75)	8 (15.38)
Widowed	10 (6.76)	4 (4.17)	6 (11.54)
Unmarried partner	22 (14.86)	12 (12.50)	10 (19.23)
Smoking status (*N* [%])			
Smoker	59 (39.86)	38 (39.58)	21 (40.38)
Non‐smoker	69 (46.62)	41 (42.71)	28 (53.85)
Former smoker	20 (13.52)	17 (17.71)	3 (5.77)
CVD (*N* [%])	23 (15.54)	15 (15.62)	8 (15.38)
Diabetes (N [%])	20 (13.51)	14 (14.58)	6 (11.54)
FMPS (mean [SD])	31.32 (19.35)	25.30 (15.37)	42.43 (21.09)
FMBS (mean [SD])	31.21 (12.61)	28.56 (11.93)	36.10 (12.48)
PPD 1–3 mm (mean [SD])	80.31 (10.53)	82.31 (9.97)	76.69 (10.65)
PPD 4–5 mm (mean [SD])	15.22 (7.84)	13.68 (7.65)	18.00 (7.46)
PPD ≥ 6 mm (mean [SD])	4.52 (5.01)	4.01 (4.89)	5.44 (5.13)
Periodontitis stage and grade (*N* [%])			
I A	12 (8.11)	11 (11.46)	1 (1.92)
I B	6 (4.05)	4 (4.17)	2 (3.85)
II A	26 (17.57)	17 (17.71)	9 (17.31)
II B	29 (19.59)	17 (17.71)	12 (23.08)
III B	41 (27.70)	27 (28.12)	14 (26.92)
III C	14 (9.46)	7 (7.29)	7 (13.46)
IV B	11 (7.43)	8 (8.33)	3 (5.77)
IV C	9 (6.08)	5 (5.21)	4 (7.69)
Periodontitis extent			
Localised	48 (32.43)	30 (31.25)	18 (34.62)
Generalised	100 (67.57)	66 (68.75)	34 (65.38)
No. of implants (mean [SD])	3 (2)	3 (2)	3 (2)

Abbreviations: CVD, cardiovascular disease; FMBS, full‐mouth bleeding score; FMPS, full‐mouth plaque score; PPD, probing pocket depth.

FMPS and FMBS were 31.32 ± 12.61 and 31.21 ± 12.61, respectively. Each participant had on average three implants (SD ±2, range 1–9).

Most implants (69%) were restored with single‐unit crowns. Regarding the prosthetic connection, 54% were screw‐retained, while the remaining were cemented restorations (Table 2).

**TABLE 2:** Implant characteristics and site‐specific clinical variables.

Variables	Overall (*n* = 459)	Peri‐implant mucositis (*n* = 258)	Peri‐implantitis (*n* = 201)
Implant distribution (*N* [%])			
Maxilla/Mandible	240 (52.29)/219 (47.71)	130 (50.39)/128 (49.61)	110 (54.73)/91 (45.27)
Site (*N* [%])			
Anterior	84 (18.30)	53 (20.54)	31 (15.42)
Bicuspid	165 (35.95)	88 (34.11)	77 (38.31)
Molar	210 (45.75)	117 (45.35)	93 (46.27)
Prosthesis (*N* [%])			
Single/Multiple	313 (68.19)/146 (31.81)	175 (67.83)/83 (32.17)	138 (68.66)/63 (31.34)
Prosthetic connection (*N* [%])			
Screwed/Cemented	248 (54.03)/211 (45.97)	151 (58.53)/107 (41.47)	104 (51.74)/97 (48.26)
Diameter (*N* [%])			
3.2 mm	27 (5.88)	14 (5.43)	13 (6.479)
3.8 mm	165 (35.95)	97 (37.60)	68 (33.83)
4.0 mm	159 (34.64)	85 (32.95)	74 (36.82)
4.2 mm	56 (12.20)	29 (11.24)	25 (12.44)
4.8 mm	52 (11.33)	33 (12.79)	21 (10.45)
Loading years (mean [SD])	6.36 (4.01)	6.48 (4.24)	6.22 (3.70)
nBOP (mean [SD])	3.41 (1.28)	2.88 (0.81)	4.10 (1.44)
REC max (mean [SD])	1.59 (1.89)	1.54 (1.88)	1.67 (1.89)
PPD max (mean [SD])	5.59 (1.66)	4.93 (1.44)	6.45 (1.55)
KT	1.82 (1.29)	1.93 (1.35)	1.68 (1.21)
SUP (*N* [%])	68 (14.81)	8 (3.10)	60 (29.85)

Abbreviations: KT, keratinised tissue height; nBOP, number of bleeding sites per implant; PPD max., maximum probing pocket depth; REC max, maximum recession; SUP, suppuration.

**FIGURE 1:**
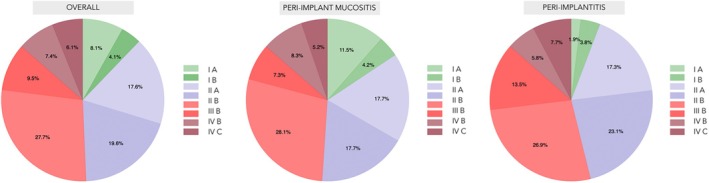
Frequency distribution of periodontitis.

Figure 1 shows the percentage distribution of periodontitis stage and grade in the study cohort. The majority of patients were classified as having Stage III Grade B (27.7%), Stage II Grade B (19.6%) or Stage II Grade A (17.6%) periodontitis. Stage I forms were less common (8.1% Grade A and 4.1% Grade B), as were the more severe Stage IV, observed in 7.4% (Grade B) and 6.1% (Grade C) of patients. Stage III Grade C was recorded in 9.5% of cases.

### 3.2 Questionnaires

#### 3.2.1 Brief‐IPQ

The results of the Brief‐IPQ, overall and according to the PID diagnosis, are reported in Table 3.

**TABLE 3:** Brief illness perception questionnaire (brief‐IPQ) and oral health impact profile 14 (OHIP‐14) scores.

Brief‐IPQ Items		Overall			Peri‐implant mucositis			Peri‐implantitis			*p*
		Median	Q1	Q3	Median	Q1	Q3	Median	Q1	Q3	
Item 1 (Consequences)	Cognitive illness	1	0	2	1	0	2.75	1	1	2	0.49
Item 2 (Timeline)		4	2	6	4	2	6	3.5	2	6	0.44
Item 3 (Personal control)		4	3	5	4	3	5	4	2.25	5	0.76
Item 4 (Treatment control)		6	5	7	6	4.25	6	6	5.25	7	0.01
Item 5 (Identity)		1	1	4	1	1	4	1.5	1	4	0.76
Item 6 (Concern)	Emotional representation	3	1	5	3	1	5	3	1	5	0.33
Item 7 (Understanding)	Illness comprehensibility	5	4	6	5	4	6	5.5	4	6	0.34
Item 8 (Emotional response)	Emotional representation	1	0	4	1	0	4	1	0	4	0.46
Sum score		28	21.25	33	27	22	31.75	29	20.25	33	0.29

OHIP‐14 Items		Overall			Peri‐Implant mucositis			Peri‐Implantitis			*p*
		Median	Q1	Q3	Median	Q1	Q3	Median	Q1	Q3	
Item 1	Functional limitation	0	0	1	0	0	1	0	0	1	0.97
Item 2		1	0	1	1	1	1	1	0	1	0.02
Item 3	Physical pain	1	1	1	1	0	1	1	1	1	0.02
Item 4		0	0	1	0	0	1	1	0	1	0.07
Item 5	Psychological discomfort	0	0	1	0	0	1	0.5	0	1	0.83
Item 6		1	0	1	1	0	1	1	1	2	0.00
Item 7	Physical disability	0	0	1	0	0	1	0	0	1	0.15
Item 8		1	0	1	1	0	1	1	0	1	0.10
Item 9	Social disability	0	0	1	0	0	1	0	0	1	0.42
Item 10		0	0	1	0	0	2	0	0	1	0.49
Item 11	Handicap	0	0	1	0	0	0.75	0	0	1	0.57
Item 12		0	0	1	0	0	1	0	0	1	0.50
Item 13		0	0	0	0	0	0	0	0	0	0.59
Item 14		0	0	0	0	0	0	0	0	0	0.45
Sum score		6	4	9	5	4	8	7	4	9	0.12

Overall, the highest scores were recorded for item 7 (‘understanding’) and item 4 (‘treatment control’) (median 5 and 6, respectively). The latter was significantly higher among patients with peri‐implantitis. The score for item 4 (‘treatment control’) was also higher than that of item 3 (‘personal control’), which had a median value of 4. Item 2 (‘timeline’) received an intermediate score. Emotional items, while scoring lower overall, showed a trend of being higher in patients with peri‐implantitis, even though this difference did not reach statistical significance. The overall median of the Brief‐IPQ sum score across the entire cohort was 28 (IQR: 21.25–33). The median was 27 in the peri‐implant mucositis group and 29 in the peri‐implantitis group, with no significant inter‐group differences.

**FIGURE 2:**
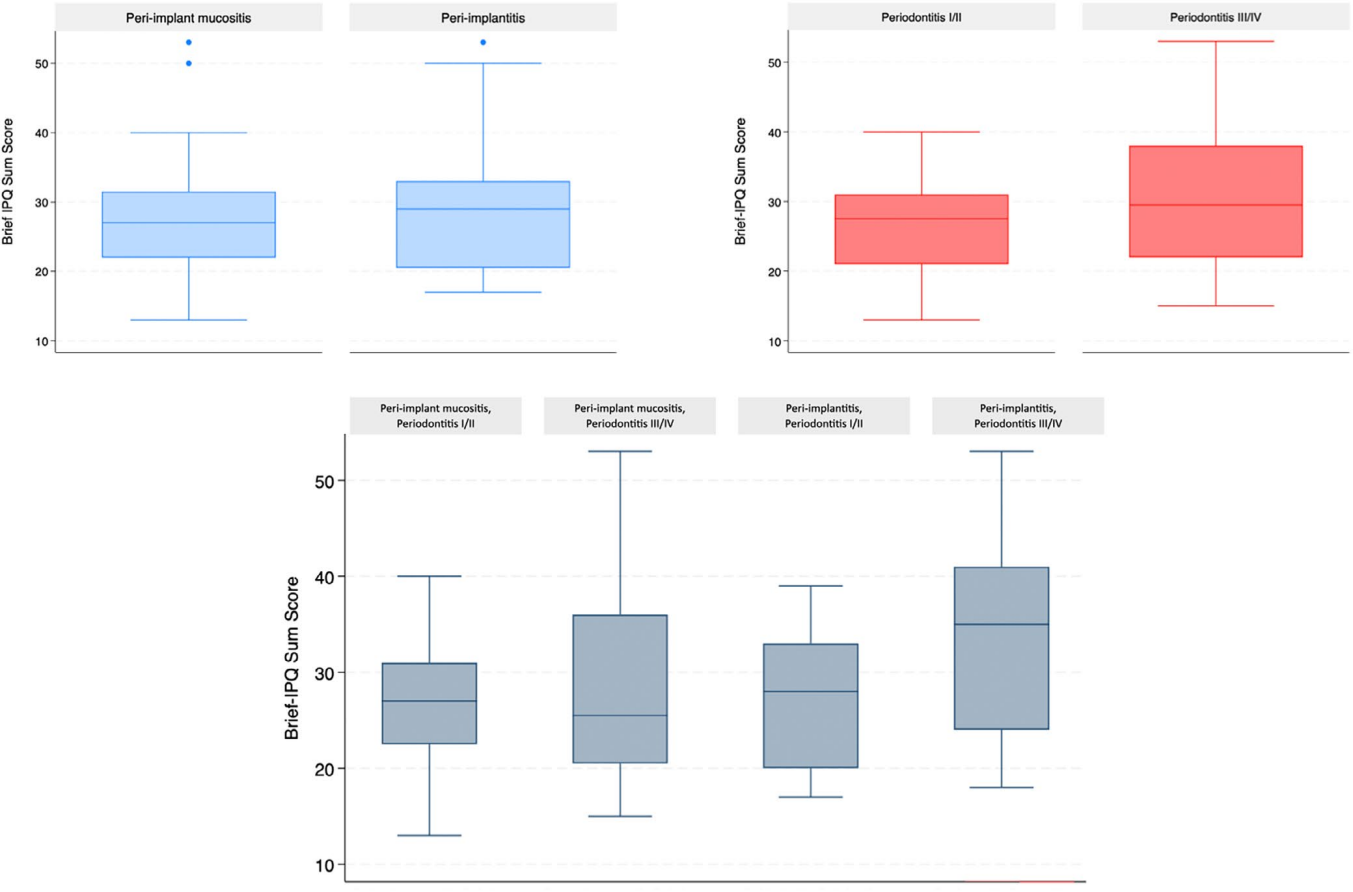
Distribution of Brief‐IPQ sum score according to the diagnosis of peri‐implant disease and periodontitis.

The box plots in Figure 2 summarise the distribution of the Brief‐IPQ sum score according to the diagnosis of PID, periodontitis and considering both clinical scenarios. A statistically significant difference was found among patients diagnosed with peri‐implantitis and stage III/IV periodontitis, who exhibited significantly higher values compared to all other groups (*p* < 0.05).

#### 3.2.2 OHIP‐14

The median total score of the OHIP‐14 questionnaire was 6 overall (Table 3), which was slightly higher in the peri‐implantitis group (median of 5 in the mucositis group and 7 in the peri‐implantitis group), although this difference was not statistically significant. Significantly higher median values were recorded in the domains of functional limitation, physical pain and psychological discomfort among patients with peri‐implantitis.

### 3.3 Logistic Multivariate Regression Model

The multivariate logistic regression model identified two variables significantly associated with higher Brief‐IPQ sum scores (> 28): having three or more implants (OR = 4.16; 95% CI: 1.81–9.57; *p* = 0.00) and a self‐reported history of periodontitis treatment (OR = 6.58; 95% CI: 2.87–15.07; *p* = 0.00) (Table 4).

**TABLE 4:** Multivariate logistic regression model.

Brief IPQ sum score (AUC = 0.8; AIC = 182.1; BIC = 200)						
LR chi^2^	Prob > chi^2^	Pseudo‐*R* ^2^				
44.41	0.00	0.22			95% CI	
Brief IPQ sum score > 28	OR	SE	Z	*p*	Lower	Higher
Peri‐implantitis	1.39	0.59	0.78	0.43	0.61	3.21
Periodontitis stage III/IV	0.74	0.39	−0.58	0.57	0.27	2.06
No. of implants ≥ 3	4.16	1.77	3.35	**0.00**	1.81	9.57
Diabetes	0.34	0.21	−1.73	0.08	0.10	1.15
Previous history of periodontitis treatment	6.58	2.78	4.46	**0.00**	2.87	15.07
Occupation						
Unemployed	2.17	1.91	0.88	0.38	0.39	12.21
Retired	0.91	0.38	−0.22	0.83	0.41	2.06
SUP (implant)	1.19	0.69	0.31	0.75	0.39	3.72
_cons	0.21	0.08	−4.03	**0.00**	0.10	0.45

*Note*: The bold font highlights the statistically significant *p*‐values.

Abbreviations: Brief IPQ, brief illness perception questionnaire; SUP (implant), presence of suppuration at implant sites.

Diabetes was associated with lower odds of having a Brief‐IPQ sum score > 28 (OR = 0.34; 95% CI: 0.10–1.15; *p* = 0.08); however, this association did not reach statistical significance.

No significant associations were found for peri‐implantitis diagnosis (OR = 1.39; 95% CI: 0.61–3.21; *p* = 0.43) and for more advanced stages of periodontitis (OR = 0.74; 95% CI: 0.27–2.06; *p* = 0.57) included in the model.

## 4 Discussion

The present study evaluates patients' perceptions of PIDs in a cohort of individuals affected by both PIDs and periodontitis using the Brief‐IPQ (Broadbent et al. 2006). Patients with peri‐implant mucositis and peri‐implantitis showed low levels of disease perception and reported a limited impact of these conditions on their OHRQoL. Most participants reported mild symptoms and low emotional involvement, and tended to underestimate the importance of their role in managing the disease.

Overall, Brief‐IPQ scores did not significantly differ between peri‐implant mucositis and peri‐implantitis patients. However, higher illness perception was observed in patients presenting with both peri‐implantitis and stage III/IV periodontitis. Additionally, having three or more implants and a history of periodontitis treatment were significantly associated with increased illness perception.

The poor perception of PIDs may be attributed to insufficient patient education during treatment planning and generally low oral health literacy (OHL), particularly in periodontally compromised individuals (Baskaradoss 2018; Wehmeyer et al. 2014). Patients often perceive dental implants as more resilient and requiring less maintenance than natural teeth, which may explain the preference for implant rehabilitation over periodontal treatment (Brunello et al. 2020; Wang et al. 2015). Furthermore, beliefs about chronic conditions can influence emotional responses and treatment adherence. Petrie and Weinman (2006) highlight the frequent omission of illness perception assessment in medical consultations. Addressing disease awareness and beliefs about causality and progression is crucial for chronic conditions, where preventive measures and maintenance are essential for therapeutic success (Petrie and Weinman 2006; Carra et al. 2023). Educating patients about the complications and failure risks associated with dental implants is a fundamental part of a primordial preventive strategy (Carra et al. 2023; Tonetti et al. 2015). Emphasising the patient's role in all treatment phases is crucial for ensuring therapy and supportive care adherence (Echeverría et al. 2019; Yao et al. 2017).

A recent study conducted on a cohort of patients with PIDs revealed that, although an increase in spontaneous discomfort was observed in the presence of PIDs compared to implants with healthy peri‐implant tissues, pain was experienced in only 7.6% of the participants (Romandini et al. 2021). The silent nature of PIDs, combined with the low illness awareness, undoubtedly makes it more difficult for patients to perceive a significant problem that would justify seeking dental care. In this scenario, the role of the clinician turns fundamental to reach an early PID diagnosis, intercepting the disease at more manageable stages.

Current evidence on periodontitis patients' psychometric profiles suggests that the perception of periodontitis is generally lower than other chronic conditions (Machado et al. 2019). Our findings suggest an even more limited representation of PIDs, as the recorded Brief‐IPQ scores were lower than those reported in studies investigating periodontitis perception using the same tool (Machado et al. 2019; Discepoli et al. 2022). However, when stratifying the data according to disease severity, the distribution of Brief‐IPQ sum scores revealed that patients with both peri‐implantitis and stage III/IV periodontitis showed significantly higher levels of perceived illness compared to all other clinical scenarios, possibly reflecting a cumulative effect. This finding may suggest that the cognitive and emotional representation of PIDs is more pronounced in patients with more advanced clinical profiles, which may be attributed to the higher disease burden experienced in these cases.

Furthermore, the findings highlight the significant role of the number of implants in shaping patients' psychometric dimensions. Specifically, having three or more implants was associated with a 4.16‐fold increase in the odds of reporting a Brief‐IPQ total score above the cohort median (28), reflecting greater illness perception within this study population. This association may suggest that patients with a higher number of implants, having been more frequently exposed to dental care, treatment planning or complications, may have developed heightened awareness and perception of disease. Moreover, patients who self‐reported a previous history of periodontitis treatment showed 6.58‐fold higher odds of reporting elevated Brief‐IPQ total scores, which may similarly reflect increased disease awareness related to prior therapeutic experience. Implant‐specific characteristics and clinical parameters did not show any significant influence in shaping patients' cognitive and emotional responses related to their illness.

Although an increase in certain domains of the OHIP‐14 was observed in patients with peri‐implantitis compared to those with peri‐implant mucositis, the overall impact of PIDs on patients' quality of life is low. The present findings are consistent with the available literature on similar psychometric parameters in patients with PIDs, which report generally modest effects on OHRQoL (Insua et al. 2017; Romandini et al. 2021). Moreover, a recent systematic review and meta‐analysis by Agnese et al. (2025) confirmed a strong association between periodontitis severity and impaired OHRQoL (Agnese et al. 2025). In our cohort, however, the perceived impact on the quality of life suggests that patients may cognitively recognise the disease burden without perceiving a proportional impact on daily functioning or well‐being. No regression analysis was performed for OHIP‐14, as this measure was used solely for descriptive purposes, to support the psychometric characterisation of the study cohort.

Some limitations should be considered when interpreting the results of this study. The inclusion of a control group free of periodontitis could have helped to isolate the specific contribution of periodontal disease to PIDs perception and extended the applicability of the findings to other PIDs populations. Moreover, although the description of patient‐reported outcomes related to quality of life was a secondary outcome, a control group free of PIDs could have made it possible to draw inferences on OHRQoL. Therefore, the present OHIP‐14 findings should be interpreted descriptively and in the context of comparisons with similar populations reported in the literature. Participants were recruited from a university hospital setting, limiting the generalisability of the findings. The results should be interpreted as associations within a cross‐sectional framework, and future longitudinal studies are needed to better understand the directionality of these associations. Additionally, information regarding previous periodontal treatment was based on patient' self‐report; therefore a certain degree of inaccuracy or misreporting cannot be excluded. Finally, while OHL was conceptually relevant to the study framework, it was not directly assessed with a specific validated instrument, which could be explored in future research.

## 5 Conclusions

The cognitive and emotional representation of PIDs appeared generally low in patients affected by both periodontitis and PIDs. However, in the presence of more advanced clinical profiles, such as the coexistence of peri‐implantitis and stage III/IV periodontitis, an *added burden* effect between the two diseases on illness perception was suggested. A higher number of implants and prior therapeutic experience are significantly associated with higher Brief‐IPQ scores, reflecting greater disease representation.

## Author Contributions

**Isabella De Rubertis:** investigation, methodology, writing – original draft. **Adriano Fratini:** methodology, formal analysis, writing – original draft. **Alice Ferrari:** investigation, writing – review and editing. **Raffaele Mirra:** conceptualisation, writing – review and editing. **Nicola Discepoli:** conceptualisation, writing – review and editing, formal analysis.

## Consent

All enrolled patients were informed about the study protocol and were asked to read and sign the informed consent form.

## Conflicts of Interest

The authors declare no conflicts of interest.

## Data Availability

The data that support the findings of this study are available from the corresponding author upon reasonable request.

## Appendix 1 Study Size

To further support the validity of the study results, a post hoc power analysis was conducted based on the final logistic regression model. In this analysis, the Brief‐IPQ total score (dichotomised at > 28) was the dependent variable, and a binary predictor of interest (number of implants ≥ 3) was coded as 1 in 47.97% of cases. The observed odds ratio was 4.16. The intercept of the model was 0.2143, corresponding to an expected probability of 0.5532 under the null hypothesis. The Nagelkerke pseudo‐*R* ^2^, calculated from a model where this binary predictor was regressed on the remaining covariates, was 0.11. Based on these parameters, and a total sample size of 148 participants, the achieved power was approximately 0.94 (calculated in G*Power, version 3.1.9.4), using a two‐tailed *Z* test for logistic regression with *α* = 0.05. This supports the adequacy of the sample in detecting associations of interest within the model.
